# Health Services Research Spending and Healthcare System Impact: Comment on "Public Spending on Health Service and Policy Research in Canada, the United Kingdom, and the United States: A Modest Proposal"

**DOI:** 10.15171/ijhpm.2017.92

**Published:** 2017-08-06

**Authors:** Morris L. Barer, Stirling Bryan

**Affiliations:** ^1^Centre for Health Services and Policy Research and School of Population and Public Health, University of British Columbia, Vancouver, BC, Canada.; ^2^Centre for Clinical Epidemiology & Evaluation and School of Population and Public Health, University of British Columbia, Vancouver, BC, Canada.

**Keywords:** Health Services Research Spending, Research Uptake, Knowledge Translation, Strategy for Patient Oriented Research (SPOR), Canada

## Abstract

The challenges associated with translating health services and policy research (HSPR) evidence into practice are many and long-standing. Indeed, those challenges have themselves spawned new areas of research, including knowledge translation and implementation science. These sub-disciplines have increased our understanding of the critical success factors associated with the uptake of research evidence into (system) practice. Engaging those for whom research evidence is likely to help solve implementation and/or policy problems, and ensuring that they are key partners throughout the research life-cycle, appear to us (based on current evidence) to be the most direct and effective paths to improved knowledge translation. In that regard, building on Canada’s recent Strategy for Patient Oriented Research (SPOR) would seem to offer considerable promise. The "modest" proposals offered by Thakkar and Sullivan seem less likely to bear fruit.

The recent paper by Thakkar and Sullivan^1^ offers a number of interesting insights, and some thoughtful suggestions. Amongst the former, perhaps the most striking is the wide disparity across three nations (the United States, the United Kingdom, and Canada) in per capita spending on healthcare system-related research, and the apparent lack of any relationship between that spending, and evidence of impact on the quality, effectiveness and efficiency of the countries’ respective healthcare systems. Not surprisingly, the United States can boast both the most expensive (per capita) healthcare system, and the highest per capita spending on system-related health research. The country with the lowest per capita research spend (UK) appears to have shown the most impact of that research spending on healthcare system performance. The authors suggest that the latter may be a product of the facts that the United Kingdom has a national healthcare system, whereas Canada’s is fragmented both by geography and sector, and that the United States is a triumph of chaos and interests over systematic design and patient focus. In fact, the United Kingdom no longer has a single healthcare system. Devolution has had real impact in terms of healthcare organization and delivery in the United Kingdom such that the National Health Service (NHS) in England looks and feels quite different to that in Scotland and Wales.^2^

We were surprised on reviewing the rank order of country health services and policy research (HSPR) spend per capita. Our prior was that the United Kingdom would be above Canada, given the major increases in the United Kingdom. health research spend over the last couple of decades. Putting aside our skepticism regarding the HSPR spend data, the apparent lack of correlation between HSPR research spending and system effectiveness or efficiency makes one of the authors’ two key recommendations rather perplexing. In particular, they suggest that these countries (and presumably others) invest a fixed percentage of healthcare spending on HSPR, and then earmark that HSPR funding to “effective HSPR investments which are instrumental in yielding better health outcomes.”^1^ We see (at least) two fundamental problems with this “modest” proposal. The first part of it seems logically inconsistent with the authors’ findings of no relationship between amounts spent on HSPR and healthcare system impact. There is no known evidence-based chain of logic that runs from a fixed percent of healthcare spending dedicated to HSPR to direct, measurable, and significant impacts on a healthcare system’s quality, effectiveness and efficiency. The second part appears more troubling still. As the authors themselves note, “[t]here is a pressing need to track the impact of increased investments in HSPR on improvements in health system performance and quality.”^1^ But if that is so (and we agree it is), how could one possibly identify which HSPR investments were effective in meeting the “better health outcomes” goals prior to making the investments and doing the evaluations of impact?

The problems do not end there. The HSPR literature is littered with shining examples of compelling evidence developed over long periods of time, pointing to particular policies that would, in all likelihood, meet the Triple Aim^3^ (to improve patient experience, population outcomes and affordability). Policy progress has not followed. In Canada, the training and deployment of nurse practitioners and advanced practice nurses,^4-6^ and the development of a national program to cover the costs of prescription medications to reduce non-compliance and overall costs^7,8^ come immediately to mind. It seems that in these, and other, areas, additional investments in HSPR would be inefficient. Rather, for such well-researched HSPR topics, new research investments might more productively focus on knowledge translation and implementation science, to help us understand and overcome the stalling of policy implementation.

Thus, the authors appear to offer a too-narrow understanding of the policy-making process (in health as elsewhere), and a rather too optimistic view of the importance of research evidence in that service. Finally, even if one were somehow able to channel that fixed percentage to areas of potential system improvement informed by suggestive evaluative evidence, there is no guarantee that the evidence will be generalizable or, even if it is so theoretically, that it will generalize in practice. For example, well-governed and managed institutions tend to be high-performing.^9,10^ But scalability has always been, and is likely to continue to be, the challenge.

The authors’ second recommendation, that a concerted effort be mounted to move Organisation for Economic Co-operation and Development (OECD) countries toward common nomenclature, indicators and measurement of HSPR is, of course, commendable and not something about which one would get much argument, from any quarter. But our observations of the difficulties of getting to common metrics and reporting standards within just one country (Canada) do not leave us optimistic about the realistic prospects for such a project, at anything beyond the most aggregate of indicators. Interprovincial (or even in some cases inter-regional) comparative HSPR in Canada continues for the most part to be a barren wasteland,^11^ requiring researchers to deal not only with lack of common nomenclature and indicators, but with multiple (and often lengthy) data access and ethics approval processes – not impossible, but the overhead costs are high. While the Canadian Institute for Health Information is well-positioned to take a leadership role in reducing these sources of friction, at the end of the day, success is completely in the hands of the individual provinces and territories, which have at best a chequered history of working together (in healthcare or any other jurisdiction – see, for example, interprovincial movement of wine and spirits, or agreement on pipelines). Not the least troublesome amongst many impediments to progress on this front is that agreeing to the comparability project in any arena opens one up to the possibility of being found to be below average (since that will, inevitably, be the fate of at least some of the jurisdictions).

As the authors note, despite substantial investments in HSPR in all three countries, we remain well short of a system, in any of the three countries, that provides comprehensive and timely information on the effectiveness and efficiency of HSPR. Building on the early work by Buxton,^12^ important progress is being made in refining the process of estimating the return on research investment.^13^ But natural experiments and purpose-built policy trials are rare, and healthcare systems are messy and dynamic, and so generally offer up very low signal to noise ratios.

The authors also note, and we endorse, the promise of the recent Canadian-developed “Strategy for Patient-Oriented Research” (SPOR). This initiative is designed to improve patient care through research that involves not only clinicians but also other key stakeholder groups including system managers and policy-makers and, importantly, patients and their families. It envisions patients being involved in identifying research priorities, and being involved throughout the research process, including in the application of results to changes in how patients experience the healthcare system. Patients are viewed as important, for example, to building or tweaking metrics for quality of life and helping to identify all relevant benefits of clinical interventions or system changes. It is still relatively early days for the SPOR initiative, so it is still too soon to conclude that it will have the impact on patient outcomes that its architects imagined (or at least dreamed of). But the recognition that patients have important perspectives to offer to the research enterprise and should, after all, be the primary focus of all health-related research, is significant and long overdue.

Building on this, and on the fact that the importance of embedding knowledge translation and implementation science into HSPR is increasingly understood,^14^ we would suggest an alternative, modest and evidence-based proposal. One can find many examples of healthcare system-improving research in all three countries mentioned by Thakkar and Sullivan.^15-18^ An element common to the vast majority of these is the involvement of system decision-makers throughout the research life-cycle.^19,20^ Yet, at least in Canada, and despite concerted efforts by the Canadian Institutes of Health Research’s (CIHR’s) Institute of HSPR, and the Canadian Health Services Research Foundation before it, much of HSPR continues to be plagued by two problems. The first is that insufficient attention is given to system relevance in the research evaluation (peer review) process. As a result, even research that is methodologically reasonable and rated as of high importance by system managers and/or policy-makers, is too often left on the cutting room floor, done in by hyper-attention to what we would coin pin-head quibbles – concerns about methods that are unlikely to have any practical impact on results; the best becomes the enemy of the potentially practically very useful. The second is that too few vehicles for truly engaging system managers and policy-makers in the research life-cycle (what CIHR refers to as “integrated knowledge translation”)^21^ exist. For example, the highly effective CIHR PHSI (Partnerships for Health System Improvement) research funding vehicle, which required collaboration between research teams and system policy-makers, was recently discontinued (or rather effectively disemboweled) by the funder.

This leads to our own modest proposal – that Canada and potentially other OECD nations build upon the foundations laid by SPOR and expand the Strategy’s current focus on patient engagement, to embrace real and effective engagement from other key stakeholders, in particular system decision/policy makers. Our proposal would imply an HSPR stream within SPOR, to ensure a strong voice for those with healthcare system management or policy responsibilities, and would thus additionally involve policy-makers and system managers throughout the process. Importantly, this would include the critical preliminary stage of identifying research priorities at a more granular level than has been the practice in the “Listening for Direction” exercises in Canada. The envisioned priorities relating to HSPR would, of course, primarily reflect policy-makers’ and managers’ immediate to medium term needs for evidence to guide system changes over which they have accountability (and the tools to initiate change and the funding to evaluate the impacts of change). Once HSPR research project or program priorities were identified, those involved in setting these priorities would become a critical part of the research teams. Complementary activities could include helping design system impact metrics that matter *to them* and in particular leading the knowledge translation and exchange process. In our view, the most effective way to increase the uptake of research evidence in the service of system change is to ensure that the research questions are important to those with responsibility for system performance. If they have ‘skin in the game’ (preferably both funding and incentives to use research results), they are more likely to want to be involved and to move research results into practice.

We should be under no illusions, however, about the difficulty of mapping research results to system change. Research evidence accumulates over time, generally requires replication to instill confidence, and can often prove fickle – last week’s revolutionary finding can turn out to be next week’s old (or alternative) news, replaced by newer and more compelling evidence. Policy-makers and managers must be cautious about embracing HSPR evidence too quickly. This makes the task of mapping HSPR evidence to particular system change – the elusive search for return on investment (ROI) – an ongoing challenge.

In sum, we would simply caution that adopting too-simple ‘fixes’ such as magic ratios of HSPR investment to system expenditures, or trying to impose or extract common metrics on wildly divergent systems, may simply end up sending good money after bad.

## Acknowledgements

The authors wish to thank Megan Ahuja for editorial and referencing support.

## Ethical issues

Not applicable.

## Competing interests

Authors declare that they have no competing interests.

## Authors’ contributions

MLB was the lead author, developing the approach to the commentary and undertaking the initial draft; SB was a contributing author.

## Authors’ affiliations

^1^Centre for Health Services and Policy Research and School of Population and Public Health, University of British Columbia, Vancouver, BC, Canada. ^2^Centre for Clinical Epidemiology & Evaluation and School of Population and Public Health, University of British Columbia, Vancouver, BC, Canada.
